# The Feasibility and Clinical Evaluation of an Immersive Augmented Reality Surgical Headset Integrated with Swept-Source Intraoperative Optical Coherence Tomography for Ophthalmic Surgery in the DISCOVER Study †

**DOI:** 10.3390/diagnostics15111394

**Published:** 2025-05-30

**Authors:** Masaharu Mizuno, Karen Matar, Reem Amine, Katherine E. Talcott, Jeffrey M. Goshe, William J. Dupps, Sumit Sharma, Asmita Indurkar, John Mamone, Jamie Reese, Sunil K. Srivastava, Justis P. Ehlers

**Affiliations:** Cole Eye Institute, Cleveland Clinic, 9500 Euclid Ave, Cleveland, OH 44195, USA; mizunom@ccf.org (M.M.); mamonej@ccf.org (J.M.);

**Keywords:** Beyeonics, surgical headset, head-mounted display (HMD), intraoperative OCT (iOCT), ophthalmic surgery, 3D surgery, heads-up-display (HUD), heads-up surgery, DISCOVER study

## Abstract

**Objectives**: to evaluate the feasibility and utility of intraoperative optical coherence tomography (iOCT) utilizing an immersive augmented reality surgical headset (Beyeonics iOCT, Beyeonics Vision Ltd., Haifa, Israel) digital visualization platform with swept-source integrated OCT in ophthalmic surgery. **Methods**: As part of the Institutional Review Board-approved prospective DISCOVER study, the Beyeonics iOCT was utilized in multiple ophthalmic surgical procedures to evaluate the feasibility and utility of iOCT with this platform. The Beyeonics iOCT is a three-dimensional surgical visualization system that utilizes a swept-source integrated OCT within the digital microscope system. Surgeon feedback on system performance and integration into the surgical workflow was gathered via a prespecified survey. **Results**: Thirteen eyes of thirteen patients were included in this study. The surgical procedures consisted of four cataract surgeries, two lamellar corneal transplants, one pterygium removal, and six vitreoretinal surgeries. Surgeons were able to successfully view and review the iOCT images within the surgical Head-Mounted Display, eliminating the need for an external display. Utility feedback from surgeons included iOCT assisting with confirming wound architecture, corneal graft orientation, and retinal structure. All surgeries were completed without reverting to a conventional microscope, and no intraoperative adverse events occurred. **Conclusions**: The new visualization platform with integrated swept-source iOCT demonstrated feasibility and potential utility in multiple ophthalmic surgical platforms. Additional research related to outcomes, ergonomics, and enhanced software analysis is needed in the future.

## 1. Introduction

The surgical microscope is an essential tool in modern ophthalmic surgery. Since Richard A. Perritt first used a binocular microscope in ophthalmic surgery in 1946, various improvements have been made to the microscope, but the basic structure of the microscope has remained largely unchanged for over half a century [1].

**Figure 1:**
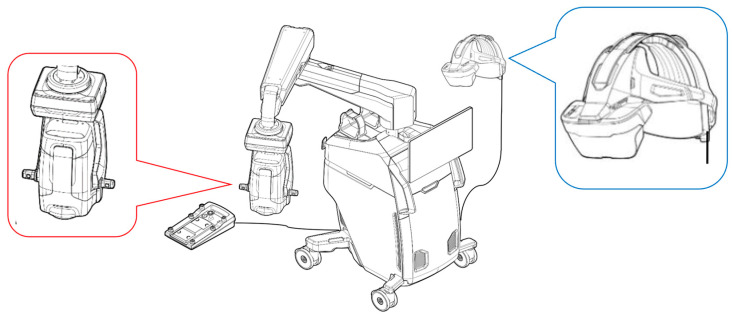
Beyeonics iOCT ophthalmic exoscope (red label) with augmented reality surgical HMD (blue label).

However, over the past decade, technological advances have led to the development of three-dimensional (3D) digital visualization platforms, known as “Heads-up-Display” (HUD), offering alternatives to standard microscopes. One approach is to allow surgeons to view the surgical field on a large 3D 4 K high-definition monitor while using passive polarized 3D glasses for stereoscopic viewing [2]. Multiple systems have utilized this approach the digital surgery, including the Zeiss ARTEVO (Zeiss, Oberkochen, Germany) and the Alcon NGENUITY (Alcon, Ft Worth, TX, USA). High-resolution digital cameras provide excellent contrast and sharpness, as well as high dynamic range capabilities [3]. The high sensitivity of the imaging processor and the adjustable gain of the digital filters allow for a reduction in the intensity of the endoscope’s lighting during surgery [4]. However, due to the distance from the display screen, the immersive viewing experience of a standard surgical microscope is not achieved [5]. Additionally, to avoid interference between the camera and the screen, the surgeon needs to look slightly to the side, which can be ergonomically unfavorable during the procedure. A new platform, Beyeonics One (Beyeonics Vision Ltd., Haifa, Israel), has been developed and is now FDA-cleared. This system provides surgeons with an immersive binocular 3D view through the use of an augmented reality Head-Mounted Display (HMD) for surgery, with the goal of offering visualization and ergonomic benefits through a pure digital display (Figure 1) [5,6,7].

The immersive augmented reality technology incorporated into the surgical headset differs fundamentally from conventional HUD systems in several aspects. First, rather than relying on an external monitor, the Beyeonics One system delivers high-resolution, stereoscopic 3D images through dual micro-OLED displays positioned within the HMD, allowing immersive visualization for the surgeon. This enables a fully immersive visual experience with enhanced depth perception and spatial awareness. Second, the system supports hands-free interaction through intuitive head gesture control, allowing the surgeon to manipulate zoom, pan, and focus in real time without interrupting the surgical workflow. Collectively, these features represent a significant evolution in surgical visualization by combining digital microscopy, intraoperative imaging, and augmented reality into a single, wearable, and surgeon-centric platform [5,6,7].

Advances in intraoperative optical coherence tomography (iOCT) technology have opened up significant possibilities for addressing various ophthalmic diseases during surgery [8]. By identifying pathological conditions and structural changes in real time, surgeons gain a deeper understanding of the anatomy, and this immediate feedback has been shown to influence surgical decision-making [9].

Recently, the Beyeonics iOCT (Beyeonics Vision Ltd., Haifa, Israel), which adds swept-source iOCT to an immersive augmented reality surgical HMD, was developed. To the best of our knowledge, devices combining these two promising technologies have not yet been evaluated. This integration represents a novel step in ophthalmic surgical visualization, combining immersive augmented reality with real-time cross-sectional imaging via iOCT within a single, fully digital intraoperative platform. The integration of real-time swept-source iOCT within the AR display allows cross-sectional imaging to be seamlessly overlaid into the surgeon’s field of view, eliminating the need to divert attention to an external screen. The purpose of this study is to describe and assess the feasibility and potential utility of integrating iOCT into a 3D digital visualization system in first-in-human surgery.

This article is an extended version of a conference presentation by Mizuno et al. [10].

## 2. Materials and Methods

This study was approved by the Institutional Review Board at Cleveland Clinic (Approval code: 14-013) and adhered to the principles of the Declaration of Helsinki. All participants provided written informed consent.

### 2.1. Subjects

The DISCOVER study is a prospective, single-center, multi-surgeon case series investigational device study evaluating the use of microscope-integrated iOCT on different platforms for ophthalmic surgery [9]. Among the participants enrolled in the DISCOVER trial, all subjects who underwent surgical intervention using a microscope-integrated swept-source iOCT system (Beyeonics iOCT) linked to an immersive augmented reality HMD in March 2024 were identified and included in this analysis.

All patients included in this analysis were enrolled under the DISCOVER study protocol, which allowed for the inclusion of adults undergoing medically indicated anterior or posterior segment ophthalmic surgery. No additional inclusion or exclusion criteria were applied beyond standard surgical indications. All patients underwent routine preoperative assessment and postoperative care according to institutional protocols. Informed consent specifically included permission for the use of digital surgical visualization systems and intraoperative imaging for research purposes.

### 2.2. Surgical Proceadure

The Beyeonics One system is a fully digital, 3D surgical visualization platform featuring dual cameras with over 8 K resolution. The surgeons performed surgery while wearing the headset, which projects a virtual image equivalent to a 4 K 3-D 55-inch OLED display 1.5 m away.

Using the Beyeonics iOCT, a microscope-integrated swept-source iOCT platform was combined with a 3D surgical visualization system to allow for the digital display of the iOCT images stream on an immersive augmented reality HMD. Surgeons with refractive errors were able to wear glasses while using the headset. The surgical headset was equipped with motion sensors that track head movements, enabling various imaging controls to be performed via gestures. The headset was connected to the digital exoscope system via a tethered cable and worn over the sterile surgical gown with the assistance of a surgical team member after sterile draping. A second headset was available for the first assistant to simultaneously visualize the surgical field and OCT images.

We performed ophthalmic surgery using Beyeonics iOCT and evaluated its utility. According to the DISCOVER study protocol, iOCT imaging was captured at various surgical milestones, based on the surgeons’ preferences. Surgeon feedback on system performance and integration into the surgical workflow was collected through a prespecified survey.

The specific procedures performed included cataract extraction with implant of intraocular lens (CE/IOL), Descemet membrane endothelial keratoplasty (DMEK), pterygium excision with conjunctival autograft, and pars plana vitrectomy using either 23- or 25-gauge instrumentation.

For CE/IOL cases, standard phacoemulsification was performed, followed by implantation of a foldable acrylic intraocular lens into the capsular bag. In DMEK procedures, donor Descemet membrane grafts were stained with trypan blue and inserted via injector, followed by manipulation for orientation and attachment. Pterygium surgery involved the excision of fibrovascular tissue and autologous conjunctival graft placement. In vitrectomy cases, core and peripheral vitrectomy was performed, followed by membrane peeling if necessary.

Throughout all procedures, iOCT was utilized at key surgical milestones (e.g., wound architecture, corneal graft orientation, retinal structure) to assess structural features in real time.

## 3. Results

### 3.1. Participants

A total of 13 eyes of 13 patients, consisting of 7 men and 6 women, with a mean age of 66.5 ± 11.8 years, were enrolled with a variety of ophthalmic diseases including cataract (*n* = 4), Fuchs endothelial corneal dystrophy (2), pterygium (1), epiretinal membrane (3), vitreous debris (2), and rhegmatogenous retinal detachment (1). Five experienced surgeons from the Cleveland Clinic Cole Eye Institute performed the surgeries. Each surgeon underwent a 10–15 min training session using a model eye to become familiar with the controls and head gestures. The control system was found to be intuitive and easy to learn.

### 3.2. Treatments

The surgical procedures consisted of CE/IOL (*n* = 4), DMEK (2), pterygium removal with conjunctival autograft (1) and 23- or 25-gauge vitrectomy (6).

**Figure 2:**
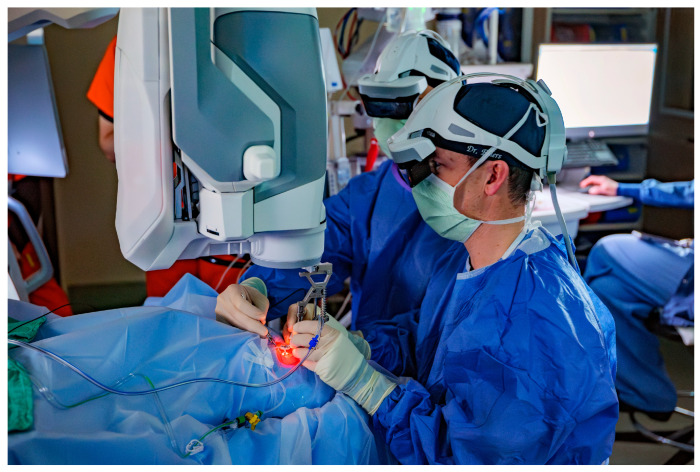
Intraoperative scene. The surgeon and first assistant can both view the same surgical images through a headset.

Surgeons reported excellent contrast and image visualization, without appreciable latency in the signal of the real-time video. Two headsets were connected, allowing the surgeon and first assistant to view the same surgical screen (Figure 2). Surgeons were able to successfully view and review the iOCT images within the surgical visor, eliminating the need for an external display for surgeon review. During vitrectomy, iOCT data were successfully visualized using both a noncontact wide-angle viewing system (BIOM, OCULUS Surgical, Port St. Lucie, FL, USA) and high-magnification contact lenses (Volk Optical, Mentor, OH, USA).

### 3.3. iOCT

**Figure 3:**
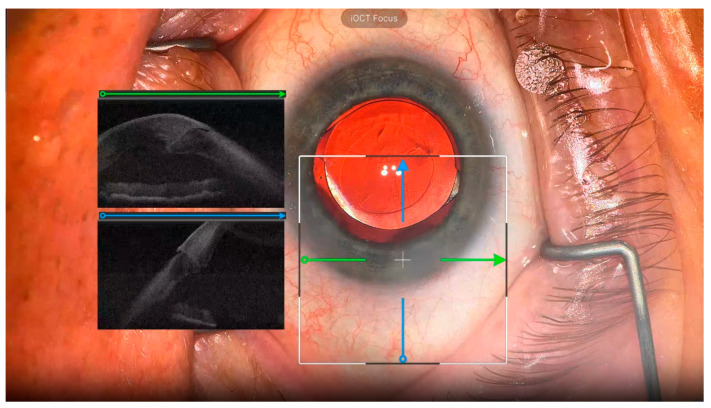
Intraoperative photograph just before the end of CE/IOL: Green arrows indicate the orientation of horizontal B-scans, and blue arrows indicate vertical B-scans. The iOCT images are displayed on the left side of the HMD, avoiding the center where the surgical procedure is being performed, so it does not obstruct the view. After hydration, the corneal edema and the cross-section of the wound architecture can be observed in detail. The extended range of the swept source system is appreciated with visualization of the cornea and iris simultaneously.

**Figure 4:**
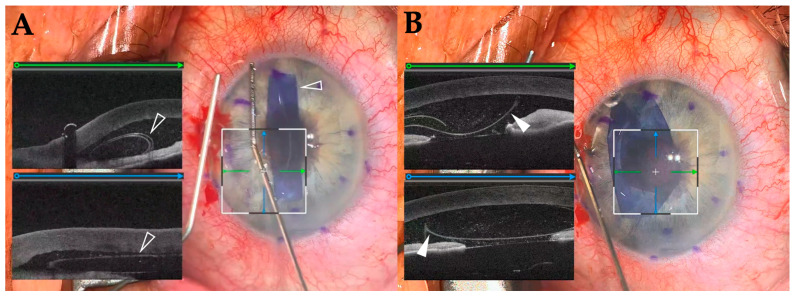
Intraoperative photograph of DMEK: Green arrows indicate the orientation of horizontal B-scans, and blue arrows indicate vertical B-scans. The scrolled graft (white open arrowheads) is visualized in the anterior chamber after insertion. iOCT enables real-time assessment of its shape and position. (**A**) After the graft is unscrolled, its orientation can be confirmed based on the characteristic curling pattern (white arrow), allowing for differentiation between the anterior and posterior surfaces without the need for tissue manipulation by S-stamp procedures (**B**).

**Figure 5:**
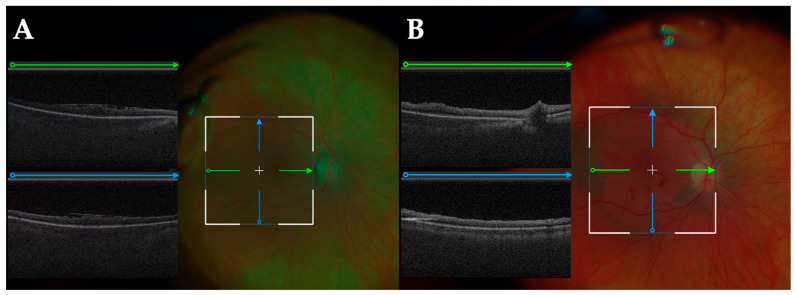
Intraoperative photograph of vitrectomy for epiretinal membrane (ERM): Green arrows indicate the orientation of horizontal B-scans, and blue arrows indicate vertical B-scans. iOCT before peeling the ERM and internal limiting membrane (ILM) helps identify the structure and extent of the ERM (**A**). After peeling (**B**), the stained ILM and the peel border become visible, while the retinal structure can be simultaneously assessed using iOCT.

The iOCT images were successfully captured and displayed in 12 out of 13 cases; in 1 case, technical issues prevented the acquisition of iOCT data. Of the 12 cases, surgeons reported receiving valuable feedback from iOCT images in 9 cases. This feedback was primarily used to visualize wound architecture and IOL position in CE/IOL (Figure 3), corneal graft orientation in DMEK (Figure 4), and retinal structure in vitrectomy (Figure 5). In one case, the surgeon reported that the surgical procedure was changed based on iOCT images; during a vitrectomy, a retinal hole was identified using iOCT after membrane peeling, leading to the addition of gas tamponade. The imaging range was sufficient to allow for visualization of the posterior lens capsule during anterior segment surgery and the choroid during posterior segment surgeries. In terms of ergonomics, all five surgeons reported that the weight of the headset resulted in an element of neck strain during surgery. Additionally, two surgeons reported that the tethering from the headset cable occasionally felt restrictive.

All surgeries were completed without reverting to a conventional microscope, and no intraoperative adverse events occurred.

## 4. Discussion

After a lull of advancements in surgical visualization, the past decade has seen a major turning point. HUD surgery is gaining more market penetration on conventional ophthalmic surgeries performed under a traditional microscope [2]. The concept of a 3D digital approach to ophthalmic surgery was reported in 1995 to improve intraoperative ergonomics [11]. Since HUD surgery uses digital signals captured by a camera, it is possible to make various real-time modifications, such as adjusting image quality, enhancing images, and converting data [3,12,13]. Image and color adjustments have enabled improved intraoperative visibility during 3D HUD surgery [14]. Retinal phototoxicity is correlated with higher light intensity [15], and it has been reported that HUD systems allow for lower light intensities for both anterior and posterior segment procedures, compared to conventional microscopes [4,16]. The HMD, the subject of this study, can adjust images similarly to a HUD, suggesting that surgery could be performed at lower light intensities.

Beyeonics One is the first ophthalmic endoscope equipped with an augmented reality HMD. It is a fully digitalized imaging platform that enables the surgeon to view a magnified 3D image of the surgical field through the headset. The device is compact and eliminates the need for a large display, potentially increasing efficiency in the operating room [2,5,6,7]. However, since the headset and exoscope are wired, the surgeon must rely on assistance to put it on after donning the surgical gown, which may be less efficient. All current HUD systems are hybrid types that allow surgery to be performed under a conventional microscope either by removing the camera or switching between the digital feed and the conventional oculars. One of the major features of Beyeonics One, however, is its use of a fully digital microscope. The exoscope uses intuitive head gesture control with auto focus, pan, zoom, and advanced control over many of the platform’s features, providing surgeons with a hands-free experience. This feature is reported to potentially improve ergonomics and reduce physical strain during lengthy procedures [5,6,7]. In this study, surgeons reported that the headset weight (approximately 500 g) resulted in neck strain, and it is possible that the physical burden could increase during long surgeries. Additionally, the tether effect of the cable was also noted at times to be restrictive. The cable connecting the HMD to the main unit was approximately 5 m in length, which was sufficient to avoid any restriction of the operating chair’s mobility. However, some surgeon discomfort may also have been attributable to the weight of the cable itself. Although not utilized in this study, the use of a more ergonomic operating chair with back support may help reduce surgeon discomfort. However, none of these factors required conversion to a conventional microscope. Future opportunities for system improvement include the ongoing efforts to transition the headset to a wireless option with a reduced weight profile. Furthermore, surgeons who are accustomed to using foot pedals may find it difficult to adapt to the head gestures. However, the system has the flexibility to use foot pedals in place of the head gestures. On the other hand, there have been some reports on the ergonomic potential of HUD, and it has been reported that using HUD reduces headaches and back pain compared to conventional microscope [17,18]. Future studies will focus on comparing light intensity and the physical burden during surgery between conventional microscopes, HUD, and HMD.

The integration of real-time intraoperative diagnosis with iOCT and 3D digital systems offers unique and exciting opportunities in the operating room, as we have published in the past DISCOVER study manuscripts [9]. In this case series, we report the first experience with ophthalmic surgery using a 3D digital HMD visualization platform in conjunction with swept-source iOCT, using the Beyeonics iOCT within the DISCOVER study.

Several previous studies have demonstrated the clinical utility of iOCT across ophthalmic and other surgical domains. For example, a systematic review by Posarelli et al. highlighted key applications and benefits of microscope-integrated iOCT in ophthalmic surgery [19]. We reported on technological advances and surgeon feedback in OCT-guided surgery using heads-up displays [20]. In the field of otologic surgery, Lee et al. explored the utility of iOCT for tympanomastoidectomy assessment [21].

While these studies established the value of iOCT and HUD systems, our study is, to our knowledge, the first to evaluate a fully immersive augmented reality HMD system integrated with swept-source iOCT in human ophthalmic surgery. The ability to visualize OCT data directly within the surgical headset—without oculars or external displays—and to control the system via intuitive head gestures represents a novel evolution in intraoperative visualization and workflow integration.

We investigated the feasibility and potential utility of integrating two state-of-the-art technologies. All surgeries were performed using the HMD without reverting to a conventional microscope, and there were no complications. In the majority of cases (92%), iOCT images were successfully acquired and displayed in real time on the augmented reality HMD. Surgeons reported that iOCT images were useful for visualizing wound structure and IOL position during CE/IOL, graft orientation during lamellar corneal transplantation (i.e., iOCT allows for real-time visualization and identification of DMEK scroll orientation, achieving this without the need for potentially harmful external markings [22]), and retinal structure before and after membrane peeling during vitrectomy.

From a surgical education perspective, HUD offers the unique advantage of allowing everyone in the room to see the same surgical screen (including iOCT images) as the surgeon. It is a great learning experience for visitors (e.g., medical students, residents) to be able to see the same surgical field as the surgeon. A previous study has demonstrated that utilizing iOCT feedback improves the ability of learning surgeons to perform ophthalmic surgical tasks (i.e., corneal suturing) with greater accuracy and precision [23]. The Beyeonics iOCT comes with two headsets, allowing both the surgeon and first assistant to share the same view, while other participants need to watch the procedure on another monitor installed on the microscope itself (Figure 1). In this regard, HUD has the advantage of allowing all participants to see the same 3D view as the surgeon by simply wearing polarized glasses. Regarding the learning curve, in this study, all surgeons were able to acquire the operation method and head gesture controls intuitively after a brief 10–15 min training session, suggesting that the device is user-friendly even for first-time HMD users.

This study has several limitations. First, the sample size was small, and no statistical analyses or quantitative comparisons were performed. As such, the results should be interpreted as an initial feasibility assessment rather than a demonstration of clinical effectiveness. Second, subjective feedback from surgeons was collected in a descriptive manner, without the use of structured scoring systems or quantitative evaluation tools. Additionally, although ergonomic challenges such as neck discomfort and cable-related restrictions were mentioned, their severity, duration, or comparison with conventional systems was not systematically assessed. Future studies are needed with larger cohorts, standardized data collection protocols, and comparative analyses with conventional microscopes and HUD systems. Key outcome measures should include surgical time, complication rates, visual outcomes, light exposure, and user satisfaction scores for both surgeons and assistants. These investigations will be critical for validating the clinical value and practical applicability of the combined iOCT and HMD platform. Another technical limitation is the absence of embedded scale bars in iOCT images. The inclusion of scale indicators would enhance the interpretability of qualitative data, and we look forward to future system upgrades that incorporate this functionality.

In the future, it is predicted that guidance using real-time imaging combined with augmented reality technology will become possible, which could be useful for positioning the corneal incision and toric IOLs. Additional intraoperative diagnostics, such as OCT angiography, could further enhance the surgical feedback platform during vitrectomy. Further software enhancements may also help measure fluid pockets and computational interface fluid volume during lamellar corneal transplantation [24], as well as changes in macular hole shape and closure speed during vitrectomy [25]. We expect more opportunities for AI-augmented integration, such as tool tracking and confirmation of surgical objectives.

## 5. Conclusions

The new visualization platform, with augmented reality surgical 3D HMD, offers advantages such as high-level immersion, high-definition images, and image control. In combination with swept-source iOCT, it may assist surgeons in performing better intraoperative diagnostics and image-guided surgery. Further studies are needed to compare this system with traditional microscope and 3D HUD approaches.

## Data Availability

Additional data available upon request.
